# *In vitro* and *in vivo* activity of the aspartic protease inhibitor CWHM-117 against *Toxoplasma gondii*

**DOI:** 10.1128/aac.00370-26

**Published:** 2026-06-22

**Authors:** Gabriela P. dos Santos, Ingrid de O. Dias, Suvajit Koley, Marvin J. Meyers, Juliana Q. Reimão

**Affiliations:** 1 Laboratory of Preclinical Assays and Research of Alternative Sources of Innovative Therapy for Toxoplasmosis and Other Sicknesses (PARASITTOS), Faculdade de Medicina de Jundiaíi 146840, Jundiaí, São Paulo, Brazil; 2 Department of Chemistry, Saint Louis University 7547, Saint Louis, Missouri, USA; The Children's Hospital of Philadelphia, Philadelphia, Pennsylvania, USA

**Keywords:** toxoplasmosis, aspartic protease inhibitors, preclinical assays, *in vitro*, mice

## Abstract

Toxoplasmosis, caused by the protozoan *Toxoplasma gondii*, affects nearly one-third of the global population and may result in severe congenital, ocular, and neurological manifestations. Current therapies are limited by toxicity, poor efficacy against chronic infection, and lack of activity against tissue cysts, highlighting the need for new therapeutic strategies. Aspartic proteases represent promising but underexplored drug targets in *T. gondii*. In this study, we evaluated the anti-*T*. *gondii* potential of a panel of aspartic protease inhibitors initially developed for inhibition of *Plasmodium*. Eleven compounds were screened *in vitro* against intracellular tachyzoites (RH strain) in human foreskin fibroblasts (HFF), and their cytotoxicity was assessed to determine EC_50_, CC_50_, and selectivity indices. Most compounds displayed micromolar activity (EC_50_ range: 1.06–75.86 µM), with CWHM-032 (=TCMDC-134675), CWHM-033 (=TCMDC-136879), and CWHM-117 emerging as the most potent inhibitors. Based on its *in vitro* selective activity, predicted pharmacokinetic and safety profile, and previously reported efficacy against experimental malaria, CWHM-117 was selected for *in vivo* evaluation. In an acute murine model of toxoplasmosis, treatment with CWHM-117 delayed mortality compared to the vehicle-treated group. In a chronic infection model, CWHM-117 significantly reduced cerebral cyst burden (*P* < 0.05), demonstrating activity against the bradyzoite stage, which remains a major therapeutic challenge. Overall, these findings indicate that aspartic protease inhibitors, particularly CWHM-117, represent promising lead compounds for the treatment of toxoplasmosis. This study supports *T. gondii* aspartic proteases as druggable targets and encourages further optimization and mechanistic studies to advance this class of compounds toward preclinical development.

## INTRODUCTION

Toxoplasmosis is a zoonotic disease caused by the obligate intracellular protozoan *Toxoplasma gondii* (1, 2). Although infection is usually asymptomatic in immunocompetent individuals, vertical transmission can lead to severe fetal complications, including spontaneous abortion, hydrocephalus, and long-term neurological sequelae (3, 4). In immunocompromised patients, particularly those with untreated HIV/AIDS or undergoing immunosuppressive therapies, *T. gondii* may disseminate to the central nervous system, resulting in life-threatening toxoplasmic encephalitis (4, 5).

Current treatment for toxoplasmosis relies primarily on the combination of pyrimethamine and sulfadiazine. These drugs are associated with significant adverse effects, including hematological toxicity and potential teratogenicity, and are mainly effective against the actively replicating tachyzoite stage (3–5). Moreover, no available therapy can eradicate the latent tissue cysts of *T. gondii*, allowing the parasite to persist in a chronic form and enabling disease reactivation under conditions of immunosuppression (5, 6). Although the clinical burden of toxoplasmosis is particularly significant in AIDS patients, epidemiological and experimental studies have shown a reduction in the incidence, morbidity, and mortality of opportunistic infections in individuals receiving antiretroviral regimens containing aspartic protease inhibitors (7–10), suggesting a potential role for this class of compounds in parasite control.

Aspartic proteases are a class of enzymes that catalyze the hydrolysis of peptide bonds and are involved in essential biological processes across a wide range of organisms. Owing to their central roles in parasite metabolism, protein maturation, invasion, and host–pathogen interactions, these enzymes have emerged as attractive targets for the development of novel anti-infective therapies (11–13). Among the most extensively studied parasite aspartic proteases are the plasmepsins of *Plasmodium* spp., the etiological agents of malaria. Although several plasmepsins were originally linked to hemoglobin degradation, subsequent studies have revealed that plasmepsin IX and plasmepsin X play central roles in parasite invasion, egress, and protein processing across multiple stages of the *Plasmodium* life cycle. Plasmepsin X is essential for the maturation of invasion-related effector proteins, whereas plasmepsin IX participates in the processing of secretory proteins required for parasite development and transmission. The druggability of these enzymes has been validated by the identification of potent dual plasmepsin IX/X inhibitors, such as WM382, which display strong antimalarial efficacy in both *in vitro* and *in vivo* models, including curative activity in murine malaria and blockade of parasite transmission (14, 15).

In *T. gondii*, seven genes encoding aspartic proteases have been identified, among which TgASP1, TgASP3, and TgASP5 are expressed during the tachyzoite stage (16, 17). TgASP3 has been shown to participate in host cell invasion by processing microneme and rhoptry proteins, whereas TgASP5 is involved in the maturation and export of parasite effector proteins that are essential for intracellular survival and virulence (17–19). Moreover, *in vivo* studies have indicated that aspartic proteases from *T. gondii* represent promising targets for both therapeutic and vaccine-based strategies (20–23).

A previous study identified aminohydantoin-derived compounds with potent antimalarial activity (24). Structure–activity relationship analyses demonstrated that these molecules act as strong inhibitors of *P. falciparum* aspartic proteases. Among them, compound CWHM-117 emerged as a promising candidate, displaying favorable oral bioavailability and significant *in vivo* antimalarial activity in murine models (12, 24). Given the phylogenetic proximity between *Plasmodium* sp. and *T. gondii*, it is possible that these compounds exhibit activity against both parasites.

Therefore, the present study aimed to evaluate a panel of antimalarial aspartic protease inhibitors (24–26) for their anti-*T*. *gondii* activity, combining *in vitro*, *in vivo*, and *in silico* approaches to investigate their antiparasitic selectivity, efficacy, predicted pharmacokinetic properties, and potential molecular targets in *T. gondii*. This work seeks to explore the repositioning potential of this compound class and contribute to the development of safer and more effective alternatives for the treatment of toxoplasmosis.

## MATERIALS AND METHODS

### Compounds

**Fig 1:**
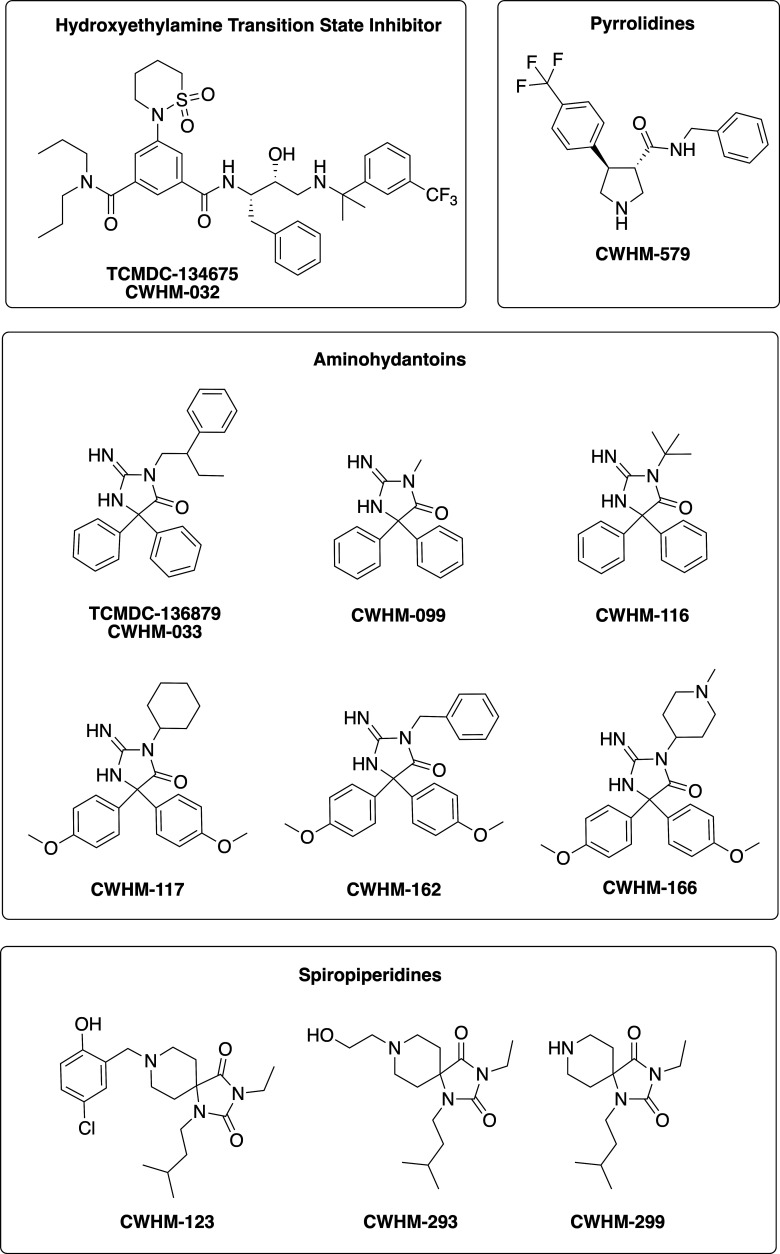
Chemical structures of the 11 aspartic protease inhibitors under study.

Meyers and coworkers previously reported on the synthesis, purification (>95% purity by HPLC), and characterization of the aspartic protease inhibitors used in this study (Fig. 1) for earlier antimalarial medicinal chemistry studies (12, 24–26). The compounds were provided by the Meyers laboratory to the Reimão laboratory for the *in vitro* studies described below. To support *in vivo* studies, compound CWHM-117 was resynthesized according to the procedure we previously described (24). After recrystallization from ethanol, CWHM-117 was obtained as a white crystalline solid (580 mg, 99% purity by HPLC; ^1^H NMR matched the literature reference). Compounds were dissolved in DMSO at 10 mM immediately prior to biological experiments.

### Identification and alignment of aspartic proteases in *P. falciparum* and *T. gondii*

Aspartic protease sequences were initially retrieved from the ToxoDB (https://toxodb.org) and PlasmoDB (https://plasmodb.org) databases (27–29). Plasmepsins from *P. falciparum* (3D7 strain) were used as reference sequences to identify potential homologs in *T. gondii* (RH strain) through amino acid sequence searches using the BLAST tool available on the VEuPathDB platform (https://veupathdb.org) (30, 31).

Selected sequences were aligned using the ClustalW algorithm via the Multiple Sequence Alignment by CLUSTALW tool available on the Genome.jp portal (https://www.genome.jp/tools-bin/clustalw) (32, 33), employing default parameters. The resulting alignments were imported into Jalview v.2.11.x (https://www.jalview.org) for visualization, editing, and generation of graphical representations (34–36).

### ADMET predictions

*In silico* predictions of absorption, distribution, metabolism, and excretion (ADME) parameters were performed using the online SwissADME tool (http://www.swissadme.ch) (37). Toxicological properties were predicted using Explorer DataWarrior v.4.7.2 (38) (OpenMolecules, https://openmolecules.org/datawarrior/download.html) and the AdmetSAR server (https://lmmd.ecust.edu.cn/admetsar2) (39). These tools were employed to identify potential pharmacokinetic liabilities and safety concerns, supporting the selection of the most promising compounds for subsequent *in vivo* assays.

### Cell and parasite maintenance

Confluent monolayers of HFF obtained from the American Type Culture Collection (ATCC, USA) were cultured in Dulbecco’s Modified Eagle Medium (DMEM; Thermo Fisher Scientific) supplemented with 10% fetal bovine serum (FBS; Thermo Fisher Scientific), 2 mM L-glutamine, and 10 μg/mL gentamicin (hereafter referred to as D10 medium), as previously described (40). Cells were previously tested and confirmed to be mycoplasma-free.

*Toxoplasma gondii* tachyzoites (RH strain, type I, clone 2F1) constitutively expressing β-galactosidase (39), were maintained in confluent HFF monolayers using DMEM supplemented with 2% FBS, 2 mM L-glutamine, and 10 μg/mL gentamicin (D2 medium). Cultures were maintained at 37°C in a humidified atmosphere with 5% CO₂ and passaged weekly, as previously described (41).

### *In vitro* anti-*T. gondii* activity assay

HFF cells were seeded into 96-well plates at a density of 5 × 10³ cells/well in 100 μL of D10 medium and incubated overnight to allow adherence. The medium was then removed and replaced with 100 μL of fresh D2 medium containing 5 × 10³ *T. gondii* RH-2F1 tachyzoites per well. Plates were incubated for 3 h at 37°C in 5% CO₂ to allow parasite invasion.

Test compounds were initially dissolved in dimethyl sulfoxide (DMSO) and subsequently diluted in D2 medium to obtain the desired concentrations. The final DMSO concentration in the assays did not exceed 1%, a concentration that showed no effect on parasite or host cell viability. Serial dilutions were prepared to generate concentration ranges from 0.39 to 100 μM. Vehicle-treated infected cells containing the corresponding concentration of DMSO were used as negative controls.

Following infection, test compounds were serially diluted in D2 medium and added to the infected monolayers. Plates were then incubated for 72 h at 37°C in 5% CO₂. Each concentration was tested in duplicate in three independent experiments. Pyrimethamine was used as a positive control in all experiments.

Parasite viability was assessed by measuring β-galactosidase activity as previously described (42). Briefly, infected cells were incubated with 100 μL of lysis buffer (100 mM HEPES, 1 mM MgSO₄, 0.1% Triton X-100, and 5 mM DTT) for 15 min at room temperature. Lysates were then mixed with 160 μL of assay buffer (100 mM phosphate buffer, pH 7.3, 102 mM β-mercaptoethanol, and 9 mM MgCl₂), followed by the addition of 40 μL of 6.25 mM chlorophenol red-β-D-galactopyranoside (CPRG). After 30 min of incubation, β-galactosidase activity was measured at 570 nm using a Thermo Scientific Varioskan LUX multimode microplate reader.

The β-galactosidase assay provides an indirect quantitative measurement of parasite proliferation, since enzyme activity correlates with the number of viable parasites expressing the reporter gene. Thus, reductions in enzymatic activity reflect inhibition of parasite growth rather than direct modulation of β-galactosidase expression.

### Cytotoxicity assay

HFF cells were seeded in 96-well plates at a density of 5 × 10³ cells per well in 100 μL of D10 medium and incubated overnight to allow adherence. Cells were then exposed to increasing concentrations of the test compounds and incubated for 72 h at 37°C in a humidified atmosphere with 5% CO₂.

Cell viability was determined using the resazurin assay as previously described (43). Briefly, resazurin (0.15 mg/mL) was prepared in phosphate-buffered saline (PBS), sterilized through a 0.22 μm membrane filter, and 10 μL was added to each well. Plates were incubated for 4 h at 37°C, and fluorescence was measured at excitation/emission wavelengths of 565/590 nm using a Thermo Scientific Varioskan LUX multimode microplate reader.

Untreated cells were used as a viability control. Cells treated with the corresponding concentration of DMSO were included as vehicle controls to ensure that the solvent did not affect cell viability. Pyrimethamine was included as a reference drug. Each concentration was tested in duplicate in three independent experiments. The selectivity index (SI) was calculated as the ratio of CC_50_ (host cell cytotoxicity) to EC_50_ (antiparasitic activity).

### Evaluation of the experimental treatment in murine models of acute and chronic toxoplasmosis

To evaluate the efficacy of the experimental treatment in the acute toxoplasmosis model, male Swiss mice (5- to 6-week old) were infected intraperitoneally with 2 × 10⁵ freshly egressed *T. gondii* tachyzoites (RH strain), as previously described (42, 44). Parasites were maintained in cell culture prior to infection and resuspended in 100 μL of sterile 0.9% saline solution for inoculation.

After infection, animals were randomly allocated into two groups (*n* = 5 per group). The non-treated group received the vehicle (0.8% NaCl, 4% DMSO, 5% PEG400, and 5% Tween 80), while the experimental group was treated with the aspartic protease inhibitor CWHM-117 at a dose of 150 mg/kg/day, administered in a total volume of 100 μL. The selected dose of 150 mg/kg/day was based on previously reported pharmacokinetic and *in vivo* efficacy studies of CWHM-117 in murine malaria models, which demonstrated adequate oral bioavailability and significant antiparasitic activity within this dosing range (24). Treatment was performed by oral gavage, starting 24 h post-infection and continued for 10 consecutive days. Treatments were administered at approximately the same time each day. Animal survival and body weight were monitored and recorded daily throughout the experimental period.

For the chronic infection model, male Swiss mice (5- to 6-week old) were orally infected with 10 brain cysts of *T. gondii* (ME-49 strain), following previously established protocols (42, 44). After infection, animals were randomly divided into two groups (*n* = 5 per group). The non-treated (NT) group received the vehicle (0.8% NaCl, 4% DMSO, 5% PEG400, and 5% Tween 80), whereas the treated group received CWHM-117 at 150 mg/kg/day in a volume of 100 μL.

Treatment was administered by oral gavage, starting on day 41 post-infection and continued for five consecutive days. On day 46 post-infection, all animals were humanely euthanized, and their brains were collected and homogenized in 5 mL of sterile 0.9% saline solution. Two 20 μL aliquots of each brain homogenate were examined under light microscopy, and the number of tissue cysts was counted for each sample, as previously described (44, 45). The mean number of cysts per animal was calculated and used for comparative analysis.

Animal experiments were approved by the Ethics Committee for Animal Experimentation (Protocol 17-2024) of the Faculdade de Medicina de Jundiaí. The research adhered to the Brazilian Guidelines for Care and Utilization of Animals from the Conselho Nacional de Controle e Experimentação Animal (CONCEA).

### Data analysis

EC_50_ and CC_50_ values were determined from sigmoidal dose–response curves using Skanlt software (Thermo Scientific). Differences in brain cyst burden between the control and treated groups were analyzed using the non-parametric Mann–Whitney test. A *P*-value < 0.05 was considered statistically significant.

## RESULTS

### The ADMET predictions suggest a favorable ADMET profile for most compounds

**Fig 2:**
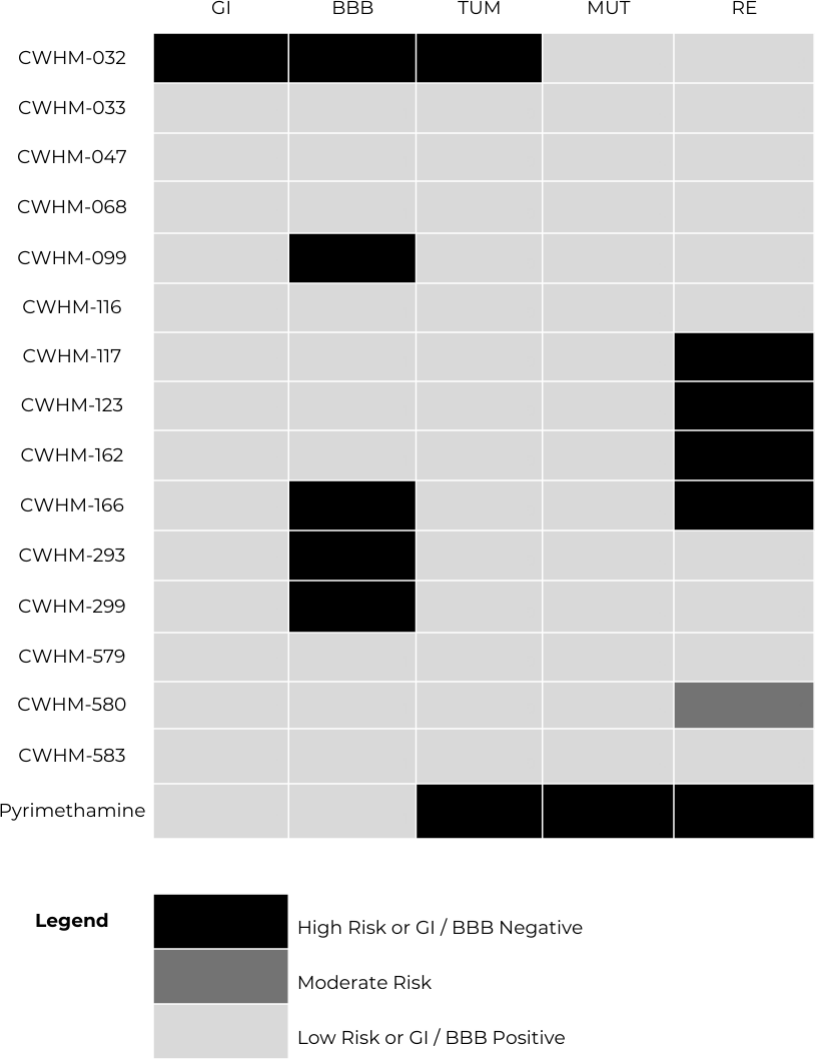
*In silico* ADMET predictions of the 11 aspartic protease inhibitors evaluated in this study. Predicted pharmacokinetic and toxicological parameters included GI absorption, BBB permeation, tumorigenicity (TUM), mutagenicity (MUT), and reproductive toxicity (RE). Predictions were generated using the SwissADME, DataWarrior, and admetSAR platforms. Favorable characteristics, including high GI absorption, BBB permeation, and low predicted toxicity risks, are represented in light gray. Unfavorable characteristics, such as low GI absorption, lack of BBB permeation, and high predicted toxicity risks, are represented in black, whereas moderate reproductive toxicity risk is shown in medium gray. Pyrimethamine was included as a reference drug for comparative purposes.

*In silico* ADMET profiling revealed that most of the evaluated aspartic protease inhibitors are predicted to exhibit high gastrointestinal (GI) absorption, except for CWHM-032, which showed low predicted GI uptake (Fig. 2). Regarding blood–brain barrier (BBB) permeation, five compounds were predicted to be BBB-permeant, suggesting potential central nervous system exposure. Toxicity predictions indicated that only compound CWHM-032 presented a tumorigenic alert. However, four molecules showed a high risk of reproductive toxicity *in silico*. Taken together, these results suggest a generally favorable predicted ADMET profile for most compounds, characterized by good oral absorption and the absence of genotoxic and carcinogenic liabilities, although the potential reproductive toxicity of some candidates should be carefully considered in subsequent stages of drug development. The results further indicate that the evaluated protease inhibitors exhibit a more favorable predicted safety profile than the reference drug pyrimethamine, which is associated with high predicted risks of mutagenicity, tumorigenicity, and reproductive toxicity.

### The plasmepsins of *P. falciparum* are homologous to the aspartic proteases 1, 3, and 5 of *T. gondii*, but have moderate similarity

To identify potential molecular targets of the aspartic protease inhibitors tested in this study, multiple sequence alignments were performed between *P. falciparum* plasmepsins and *T. gondii* aspartic proteases. The analyses revealed that plasmepsins from the *P. falciparum* 3D7 strain exhibit moderate homology with three *T. gondii* aspartic proteases from the RH strain: TgASP1, TgASP3, and TgASP5.

**Fig 3:**
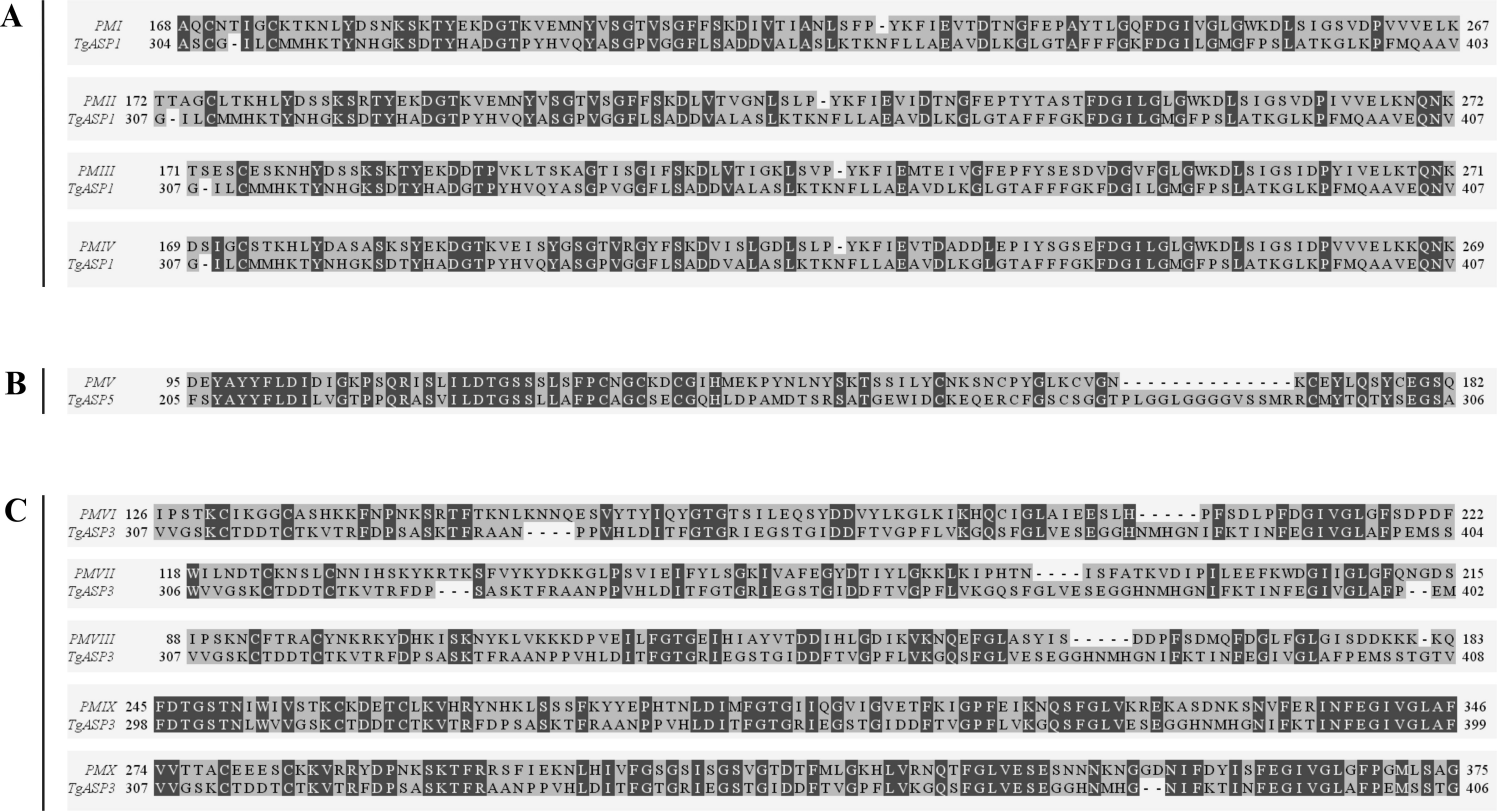
Multiple sequence alignment of *P. falciparum* plasmepsins and *T. gondii* aspartic proteases. (**A**) Plasmepsins I–IV from *P. falciparum* aligned with TgASP1 from the RH strain of *T. gondii*. (**B**) Plasmepsin V aligned with TgASP5 from the RH strain of *T. gondii*. (**C**) Plasmepsins VI–X aligned with TgASP3 from the RH strain of *T. gondii*. The numbers at the N- and C-termini of each sequence correspond to the amino acid positions in the full-length proteins, and dashes (–) represent alignment gaps. The color gradient reflects the level of residue conservation, with darker tones indicating higher identity among aligned sequences.

As illustrated in Fig. 3, distinct groups of plasmepsins aligned preferentially with specific *T. gondii* proteases. Plasmepsins I, II, III, and IV showed homology with TgASP1 (Fig. 3A), while plasmepsin V was identified as the homolog of TgASP5 (Fig. 3B). In contrast, plasmepsins VI, VII, VIII, IX, and X are aligned with TgASP3 (Fig. 3C). Conserved regions among these proteins are highlighted in darker tones in the alignment, indicating segments of higher amino acid conservation, which may correspond to preserved structural or functional motifs.

The percentage identity and similarity values obtained from the BLAST alignments are summarized in Table 1. Overall, identity values ranged from 26% to 49%, while similarity values ranged from 44% to 68%, indicating a moderate level of conservation between the proteins. Among the analyzed plasmepsins, members of the VI–X group exhibited the highest degree of conservation relative to TgASP3, with plasmepsin X showing the highest identity (49%) and similarity (68%). These findings suggest that TgASP3 is the most closely related *T. gondii* protease to several *P. falciparum* plasmepsins and may represent a primary molecular target of the investigated inhibitors.

**TABLE 1:** Identity and similarity between the aspartic proteases of *P. falciparum* and *T. gondii*^*a*^

Plasmepsin from *P. falciparum*	*T. gondii* homologous protease	Identity	Similarity
I	TgASP1	119/408 (29%)	186/408 (46%)
II	TgASP1	116/394 (29%)	180/394 (46%)
III	TgASP1	102/395 (26%)	174/395 (44%)
IV	TgASP1	99/361 (27%)	159/361 (44%)
V	TgASP5	136/475 (29%)	229/475 (48%)
VI	TgASP3	113/355 (32%)	169/355 (48%)
VII	TgASP3	104/401 (26%)	185/401 (46%)
VIII	TgASP3	116/339 (34%)	185/339 (55%)
IX	TgASP3	145/379 (38%)	216/379 (57%)
X	TgASP3	164/335 (49%)	229/335 (68%)

^*a*^ Values obtained from amino acid sequence alignment performed using the BLAST tool available on the VeuPathDB portal (https://veupathdb.org). Identity indicates the percentage of identical amino acid residues between the aligned sequences, while similarity considers residues with similar chemical properties. The differences observed in identity and similarity values suggest structural and functional variations between homologous proteases, with some showing greater conservation (higher values) than others.

### Two evaluated compounds demonstrated selective anti-*T. gondii* activity

The *in vitro* activity of aspartic protease inhibitors was evaluated against *T. gondii* tachyzoites (RH strain, clone 2F1), and their cytotoxicity was assessed in HFFs. The tested compounds exhibited heterogeneous profiles in terms of potency and selectivity. Most compounds demonstrated micromolar anti-*T. gondii* activity, with EC_50_ values ranging from 1.06 (CWHM-033) to 75.86 µM (CWHM-116), and CC_50_ values between 12.02 (CWHM-033) and >100 µM (CWHM-293 and CWHM-299) (Table 2).

**TABLE 2:** Anti-*T. gondii* activity and *in vitro* cytotoxicity of aspartic protease inhibitors under study

Compound	HFF	*T. gondii*	SI^*c*^
	CC50 (µM) ±SD^*a*^	EC50 (µM) ± SD^*b*^	
CWHM-032	12.37 ± 1.19	1.24 ± 0.13	9.97
CWHM-033	12.02 ± 2.38	1.06 ± 0.01	11.33
CWHM-099	84.80 ± 9.38	19.67 ± 5.83	4.31
CWHM-116	99.07 ± 0.03	75.86 ± 1.46	1.3
CWHM-117	14.30 ± 0.03	2.00 ± 0.36	7.15
CWHM-123	31.67 ± 8.27	5.15 ± 1.06	6.14
CWHM-162	13.64 ± 2.06	3.11 ± 0.42	4.38
CWHM-166	30.64 ± 2.43	6.67 ± 0.44	4.69
CWHM-293	>100	>100	ND
CWHM-299	>100	57.27 ± 14.71	>1.73
CWHM-579	15.8 ± 2.08	3.45 ± 0.40	4.57
Pyrimethamine^*d*^	118.60 ± 23.48	0.26 ± 0.08	453.84

^*a*^ Cytotoxic concentration 50% (CC_50_) against HFF cell line.

^*b*^ Effective concentration 50% (EC_50_) against *T. gondii* intracellular tachyzoites (RH strain, clone 2F1).

^*c*^ Selectivity Index (SI) against *T. gondii* intracellular tachyzoites, given by the ratio between CC50 and EC50.

^*d*^ Pyrimethamine (PYR) was used as a reference drug. SD: standard deviation. ND: not determined. Results obtained from duplicates in three independent experiments.

Among the evaluated compounds, CWHM-032, CWHM-033, and CWHM-117 were the most potent, displaying EC_50_ values of 1.24 ± 0.13 µM, 1.06 ± 0.01, and 2.00 ± 0.36 µM, respectively. However, these compounds also showed relatively higher cytotoxicity toward HFF cells (CC_50_ = 12.37 ± 1.19 µM for CWHM-032, 12.02 ± 2.38 for CWHM-033, and 14.30 ± 0.03 µM for CWHM-117). Despite this, their selectivity indices were close to or above 7, indicating preferential activity toward the parasite when compared with host cells.

Compounds CWHM-123, CWHM-162, CWHM-166, and CWHM-579 also exhibited promising anti-*T. gondii* activity, with EC_50_ values in the low micromolar range (3.11–6.67 µM) and moderate selectivity indices, ranging from 4.38 to 6.14. In contrast, compounds such as CWHM-116, CWHM-293, and CWHM-299 displayed low potency and poor selectivity, with EC_50_ values above 50 µM and SI values close to or below 2, suggesting limited potential as anti-*T. gondii* candidates.

As expected, the reference drug pyrimethamine exhibited high anti-*T. gondii* potency (EC_50_ = 0.26 ± .08 µM) combined with low cytotoxicity (CC_50_ = 118.00 ± 23.48 µM), resulting in a markedly higher selectivity index (SI = 453.84) when compared with the tested compounds.

Overall, these results highlight CWHM-032, CWHM-033, and CWHM-117 as the most active aspartic protease inhibitors evaluated here *in vitro*, while compounds CWHM-123, CWHM-162, CWHM-166, and CWHM-579 represent promising scaffolds for further optimization due to their favorable balance between antiparasitic activity and host cell viability. Based on its *in vitro* selective activity, predicted pharmacokinetic and safety profile, and previously reported efficacy against experimental malaria, CWHM-117 was selected for subsequent *in vivo* evaluation.

### Compound CWHM-117 demonstrated *in vivo* activity against *T. gondii* in both acute and chronic murine models of toxoplasmosis

**Fig 4:**
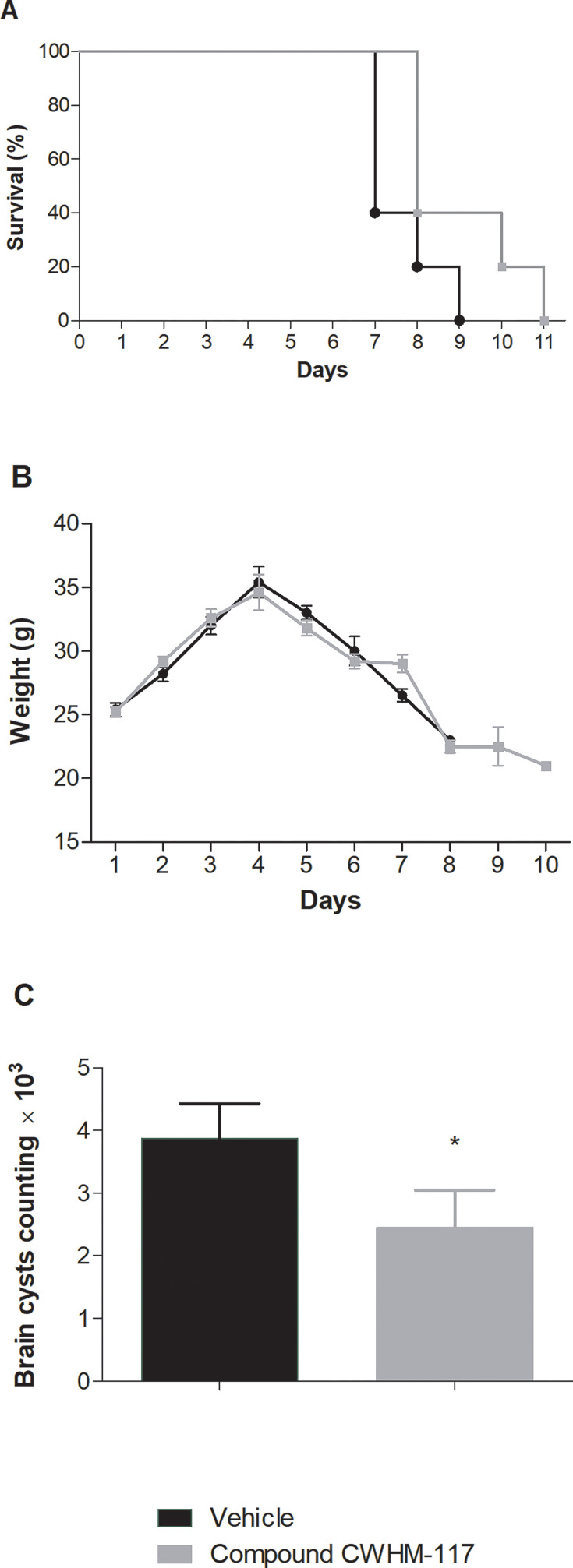
*In vivo* efficacy of compound CWHM-117 in acute and chronic murine models of toxoplasmosis. (**A**) Kaplan–Meier survival curve of Swiss mice intraperitoneally infected with the RH strain of *T. gondii* (2 × 10⁵ tachyzoites/animal) and treated orally with CWHM-117 (150 mg/kg/day) or vehicle (0.8% NaCl, 4% DMSO, 5% PEG400, and 5% Tween 80) for 10 consecutive days, starting 24 h post-infection. (**B**) Daily body weight variation of infected animals during the acute infection experiment, expressed as a percentage relative to the initial body weight. (**C**) Brain cyst burden in Swiss mice orally infected with the ME-49 strain of *T. gondii* (10 cysts/animal) and treated orally with CWHM-117 (150 mg/kg/day) or vehicle for five consecutive days, starting on day 41 post-infection. Animals were euthanized on day 46 post-infection, and tissue cysts were quantified by light microscopy in brain homogenates. Data are expressed as the mean number of cysts per animal. Statistical significance was determined using the Mann–Whitney test (*P* < 0.05 versus vehicle-treated group; *n* = 5 animals per group).

In the acute infection model, mice were challenged with the highly virulent RH strain (2 × 10⁵ tachyzoites/animal), and oral treatment with compound CWHM-117 (150 mg/kg/day) was initiated 24 h post-infection and continued for 10 consecutive days. Animals in the vehicle-treated group succumbed to infection between days 7 and 9 post-infection, whereas treatment with compound CWHM-117 extended survival, with animals surviving up to day 11 (Fig. 4A), indicating a delay in mortality associated with acute toxoplasmosis. Importantly, daily monitoring of body weight revealed no significant weight loss in treated animals compared to the vehicle group (Fig. 4B), suggesting that compound CWHM-117 was well tolerated at the administered dose.

In the chronic infection model, mice were infected with 10 cysts of the cystogenic ME-49 strain and, after 40 days, received oral treatment with compound CWHM-117 (150 mg/kg/day) for five consecutive days. On day 46 post-infection, brain cyst burden was quantified. Mice treated with compound CWHM-117 exhibited a marked reduction in the number of brain cysts when compared to the vehicle-treated group (Fig. 4C), corresponding to a statistically significant decrease in parasite burden (*P* < 0.05). These findings demonstrate that compound CWHM-117 not only delays mortality in acute toxoplasmosis but also significantly reduces cerebral cyst load during chronic infection, highlighting its potential as a promising candidate for further development as an anti-*T. gondii* agent.

## DISCUSSION

Proteolysis is an essential biological process involved in protein turnover, nutrient acquisition, signal transduction, and activation or inactivation of key regulatory proteins. This process is mediated by proteases, a broad class of enzymes that catalyze the hydrolysis of peptide bonds in protein substrates. Proteases are commonly classified according to their catalytic mechanism, which depends on the nature of the active-site residue or cofactor involved in peptide bond cleavage. The most extensively studied catalytic classes include cysteine, serine, aspartic, and metalloproteases (46–50).

In the context of drug discovery, aspartic proteases have emerged as particularly attractive molecular targets for the treatment of infectious diseases caused by protozoa and viruses (47–50). Notably, aspartic protease inhibitors have been successfully developed and widely used as antiretroviral agents in HIV therapy and have also attracted significant attention in antimalarial drug discovery (8, 11, 12, 17, 25, 26, 50). In *P. falciparum*, aspartic proteases—also known as plasmepsins—play essential roles in parasite survival and proliferation, including hemoglobin degradation, protein export and processing, as well as invasion- and egress-related events (12, 13, 51, 52).

Given the phylogenetic proximity and shared evolutionary ancestry between *P. falciparum* and *T. gondii* (17, 18, 46), it is reasonable to hypothesize that inhibitors developed against plasmepsins may also affect homologous aspartic proteases in *T. gondii*. Through amino acid sequence alignment of the ten known plasmepsins from *P. falciparum* (strain 3D7), we identified three homologous aspartic proteases in *T. gondii* (strain RH): TgASP1, TgASP3, and TgASP5. Although *T. gondii* aspartic proteases represent promising molecular targets, their biological roles and therapeutic potential remain insufficiently explored. Therefore, the present study investigated the efficacy of antimalarial aspartic protease inhibitors against *T. gondii*.

TgASP1, TgASP3, and TgASP5 are highly expressed during the tachyzoite stage and perform distinct yet essential functions in parasite protein maturation and intracellular survival (17, 18, 53). TgASP1 is a coccidian-specific protease identified in *T. gondii* and *Neospora caninum* (16, 53). It localizes near the inner membrane complex (IMC), a cytoskeletal structure that provides support and organization during daughter cell formation (16, 18). However, gene knockout experiments have demonstrated that TgASP1 is dispensable for parasite survival and is not essential for IMC biogenesis (16). These findings suggest that TgASP1 is unlikely to be the primary molecular target of the compounds tested in this study, as its inhibition does not critically impair *T. gondii* viability.

In contrast, TgASP3 plays a central role in the maturation of microneme and rhoptry proteins—organelles that are indispensable for host cell invasion and parasite egress (17). Parasite strains lacking TgASP3 display defective secretion of these organelles, ultimately disrupting the lytic cycle of *T. gondii* (54). Previous studies demonstrated that the aspartic protease inhibitor 49c directly targets TgASP3, impairing both invasion and egress of the parasite from host cells (17). These observations position TgASP3 as a highly promising therapeutic target, and we hypothesize that this protease is one of the main targets inhibited by the compounds investigated in the present study.

TgASP5 is an aspartic protease localized in the Golgi apparatus and plays a crucial role in the processing of dense granule proteins, which act as important virulence factors (54, 55). TgASP5 is also involved in parasitophorous vacuole formation and in the biogenesis of the cyst wall in bradyzoites (55). *T. gondii* strains lacking TgASP5 exhibit enlarged residual bodies, accumulation of dense vesicles within the cyst matrix, and thinner cyst walls. These structural defects result in a marked reduction—up to sevenfold to eightfold—in the number of brain cysts in infected mice (55). These findings support the hypothesis that inhibition of TgASP5 may contribute significantly to the reduction in brain cyst burden observed in our *in vivo* chronic toxoplasmosis model.

Although the aspartic proteases of *T. gondii* and *P. falciparum* are homologous, a notable difference in amino acid sequence length is evident. Our sequence alignment revealed that *T. gondii* aspartic proteases are considerably longer than their *P. falciparum* counterparts, reflecting possibly distinct evolutionary pressures and biological requirements (17, 18, 52, 56). These structural differences likely arose from gene insertions and/or deletions that occurred during parasite evolution. Nevertheless, several conserved regions are preserved, particularly within the TgASP3-associated group, suggesting structural and potentially functional similarities between these enzymes.

As previously reported, knockdown of plasmepsin IX (PMIX) and plasmepsin X (PMX) in *P. falciparum* leads to the accumulation of abnormal schizonts, impaired parasite egress, and reduced erythrocyte invasion (12). Consistently, microscopy-based assays showed that parasites treated with CWHM-117 and CWHM-032 display phenotypes similar to those observed upon PMX knockdown, including defective egress and failure to invade new erythrocytes (12). In *T. gondii*, sequence alignment analyses revealed that TgASP3 shares up to 68% amino acid similarity with PMIX and PMX, supporting the hypothesis that CWHM-117 and CWHM-032 may also target TgASP3 and interfere with parasite egress and host cell invasion through related mechanisms. Moreover, considering the conservation of catalytic motifs among aspartic proteases, it is plausible that the inhibitors evaluated here exert their antiparasitic effects through the simultaneous inhibition of more than one *T. gondii* aspartic protease.

The aspartic protease inhibitors evaluated here were originally derived from compounds active against human β-secretase (BACE), an aspartic protease associated with Alzheimer’s disease (24). Due to the structural similarity between BACE and *P. falciparum* plasmepsins—particularly Plasmepsin V—structural modifications were introduced in BACE inhibitors to redirect their activity toward parasite proteases (25). Among the resulting compounds, CWHM-117 emerged as the most promising antimalarial candidate and was subsequently evaluated *in vivo*, using the classical 4-day suppressive test in *Plasmodium chabaudi*–infected mice. In that study, oral administration of CWHM-117 resulted in a dose-dependent reduction in parasitemia, with complete elimination achieved at 300 mg/kg. Furthermore, doses of 150 or 300 mg/kg successfully suppressed infection in 10 out of 12 treated animals, demonstrating potent antimalarial activity (24).

Among the analyzed plasmepsins, members of the VI–X group exhibited the highest degree of conservation relative to TgASP3, with plasmepsin X showing the greatest identity and similarity. In light of the reported plasmepsin X inhibitory activity of CWHM-117 (24), these observations suggest that TgASP3 may represent a potential molecular target of the investigated compounds and provide a plausible explanation for the *in vitro* anti-*T. gondii* activity observed, thereby supporting the rationale for evaluating CWHM-117 in *T. gondii*–infected mice.

CWHM-117 was then selected for evaluation in both acute and chronic models of toxoplasmosis. In the acute infection model, treatment prolonged survival compared to control animals, while daily monitoring of body weight indicated good tolerability. In the chronic model, treatment resulted in a statistically significant reduction in the number of brain cysts, a key marker of disease persistence and severity.

Although CWHM-033 exhibited a slightly higher selectivity index *in vitro*, the selection of CWHM-117 for *in vivo* evaluation was based on multiple complementary criteria. In addition to its potent anti-*T. gondii* activity, CWHM-117 presented a favorable predicted pharmacokinetic profile in the *in silico* ADMET analysis and had previously demonstrated oral bioavailability and *in vivo* efficacy in murine malaria models (24). These characteristics supported its prioritization as the most suitable candidate for *in vivo* proof-of-concept evaluation in the present study.

Previously reported pharmacokinetic data for compound CWHM-117 supported the dose selection employed in the present *in vivo* studies. Oral administration of CWHM-117 at 100 mg/kg once daily resulted in measurable and sustained plasma exposure, with concentrations of 2,790 ng/mL at 1 h, 3,393 ng/mL at 6 h, and 575 ng/mL at 24 h after the last dose on day 3 of treatment (24). These findings indicate that the compound is orally bioavailable and capable of maintaining plasma levels over a 24-h interval, consistent with a once-daily dosing regimen. Based on this exposure profile and aiming to ensure therapeutically relevant systemic concentrations during the course of infection, a slightly higher dose (150 mg/kg/day) was selected in the present study to maximize efficacy while remaining within a range previously shown to achieve adequate bioavailability *in vivo*.

Although CWHM-117 displays higher potency against *Plasmodium* spp., as previously reported in antimalarial studies (24–26), it nevertheless exhibited relevant anti-*T. gondii* activity in the present work, including effects on the chronic stage of infection. The difference in efficacy between the two parasites can be attributed, at least in part, to structural and functional divergences between *P. falciparum* plasmepsins and *T. gondii* aspartic proteases. While the repurposing of antimalarial compounds represents a promising strategy, the rational design of new molecules specifically tailored to inhibit *T. gondii* aspartic proteases may further improve selectivity, potency, and therapeutic efficacy for toxoplasmosis.

Although sequence similarity and previously reported functional studies support TgASP3 as a potential molecular target of these inhibitors, direct biochemical validation of TgASP3 inhibition was beyond the scope of the present study. Therefore, future investigations employing genetic or enzymatic approaches will be required to confirm the precise molecular targets and mechanisms underlying the observed antiparasitic effects.

It is also important to note that the *in vivo* experiments were designed as an initial proof-of-concept evaluation of antiparasitic activity. Future studies, including additional experimental controls and larger sample sizes, will be important to further validate the therapeutic potential of this compound.

Taken together, these results support the repositioning of aspartic protease inhibitors as a promising strategy for the treatment of toxoplasmosis and highlight *T. gondii* aspartic proteases as druggable targets. Future studies should focus on structure-based rational design to increase selectivity toward TgASP3 and TgASP5, as well as on pharmacokinetic optimization and combination therapy approaches to enhance efficacy and minimize potential toxicity. Additionally, further investigations into the molecular mechanism of action will be essential to advance these compounds toward preclinical development.
